# Liver mitochondrial dysfunction is reverted by insulin-like growth factor II (IGF-II) in aging rats

**DOI:** 10.1186/1479-5876-9-123

**Published:** 2011-07-28

**Authors:** Maria Garcia-Fernandez, Inma Sierra, Juan E Puche, Lucia Guerra, Inma Castilla-Cortazar

**Affiliations:** 1 Department of Physiology, School of Medicine, University of Málaga, 29071 Málaga, Spain; 2 Department of Medical Physiology, CEU-San Pablo University School of Medicine Institute of Applied Molecular Medicine (IMMA) Boadilla del Monte, 28668 Madrid, Spain

## Abstract

**Background:**

Serum IGF-I and IGF-II levels decline with age. IGF-I replacement therapy reduces the impact of age in rats. We have recently reported that IGF-II is able to act, in part, as an analogous of IGF-I in aging rats reducing oxidative damage in brain and liver associated with a normalization of antioxidant enzyme activities. Since mitochondria seem to be the most important cellular target of IGF-I, the aim of this work was to investigate whether the cytoprotective actions of IGF-II therapy are mediated by mitochondrial protection.

**Methods:**

Three groups of rats were included in the experimental protocol young controls (17 weeks old); untreated old rats (103 weeks old); and aging rats (103 weeks old) treated with IGF-II (2 μg/100 g body weight and day) for 30 days.

**Results:**

Compared with young controls, untreated old rats showed an increase of oxidative damage in isolated mitochondria with a dysfunction characterized by: reduction of mitochondrial membrane potential (MMP) and ATP synthesis and increase of intramitochondrial free radicals production and proton leak rates. In addition, in untreated old rats mitochondrial respiration was not blocked by atractyloside. In accordance, old rats showed an overexpression of the active fragment of caspases 3 and 9 in liver homogenates. IGF-II therapy corrected all of these parameters of mitochondrial dysfunction and reduced activation of caspases.

**Conclusions:**

The cytoprotective effects of IGF-II are related to mitochondrial protection leading to increased ATP production reducing free radical generation, oxidative damage and apoptosis.

## Background

The reduced activity of the GH-IGFs axis leads to a condition known as the somatopause [1], which is characterised by a decrease in lean body mass and an increase in adipose mass, osteopenia, muscle atrophy, reduced exercise tolerance and changes in the plasma lipoprotein profile [2]. These alterations are similar to those observed in younger adults with GH deficiency [3]. Understanding that aging is an unrecognized condition of "IGF-I deficiency", we have recently reported that IGF-I replacement therapy restores many age-related changes increasing testosterone levels and serum total antioxidant capability and reducing oxidative damage in brain and liver [4]. This cytoprotective (neuroprotective and hepatoprotective) activity of IGF-I in aging rats is related to mechanisms of mitochondrial protection including normalization of the potential membrane and ATP synthesis and reduction of intramitochondrial free radicals production [5].

More recently we have reported that IGF-II is able to act, in part, as an analogous of IGF-I in aging rats inducing neuroprotection and hepatoprotection without increasing testosterone levels [6].

Mitochondria are specially sensitive to oxidative damage in the pathogenesis of disease and aging [7,8]. Normal mitochondrial function is a critical place in maintaining cellular homeostasis because mitochondria produce ATP and they are the major intracellular source of free radicals. Intracellular or extracellular insults converge on mitochondria [9] and induce a sudden increase in permeability of the inner mitochondrial membrane the so-called mitochondrial membrane permeability transition (MMPT). The MMPT is caused by the pores opening in the inner mitochondrial membrane, dissipation of proton gradient, matrix swelling and outer membrane rupture [8-12]. The MMPT is an endpoint to initiate cell death because the pore opening lead to the release of the mitochondrial cytochrome c activating the apoptotic pathway of caspases.

One of the most sensitive points for the pore opening is the adenosine nucleotide translocator (ANT) [10]. Since mitochondria are one of the most important cellular targets of IGF-I, the aim of this work was to investigate if the cytoprotective properties of IGF-II therapy are also related to mitochondrial protection.

Recently we have characterized the mitochondrial dysfunction in aging rats in a similar protocol [5]. In the present work, the following parameters were studied: mitochondrial membrane potential, intramitochondrial Reactive Oxygen Species (ROS) production, ATP synthesis, oxygen consumption, proton leak rates, vulnerability of pore opening and caspases activation [10-16] in young controls, untreated old rats and old rats treated with IGF-II, at low doses, for 30 days.

## Materials and methods

### Animals and experimental design

All experimental procedures were performed in compliance with *The Guiding Principles for Research Involving Animals *[17]. Healthy male Wistar rats, 17 weeks old (wk), were used in this protocol as young controls (group yCO, n = 6), and healthy male Wistar rats of 103 wk old were randomly assigned to receive either saline (0.5 mL, group O, n = 6) or recombinant human IGF-II (Lilly Laboratories, Madrid, Spain) subcutaneously (2 μg IGF-II/100 g body weight and day in 0.5 mL of saline in two divided doses, group O+IGF-II, n = 6) for 30 days.

Both food (standard semipurified diet for rodents; B.K. Universal, Sant Vicent del Horts, Spain) and water were given *ad libitum*. Rats were housed in cages placed in a room with a 12-h light, 12-h dark cycle, and constant humidity and temperature (20°C).

In the morning of the 31st day, rats were killed by decapitation, and the liver was dissected. Fresh liver was used to isolate mitochondria and to perform mitochondrial function tests using flow cytometry [4,11].

Samples were obtained simultaneously from young, old-untreated rats, and old rats treated with IGF-II to have paired data.

### Isolation of liver mitochondria

Liver mitochondrial fraction was prepared according to the method described by Schneider and Hogeboom with modifications [12]. Liver samples were homogenized (1:10 wt/vol) in an ice-cold isolation buffer containing sucrose 0.25 M and 0.1% BSA buffered (pH 7.4) with Tris-HCl 10 mM, and the isolation medium was identical without BSA or EDTA. The protein concentration was measured using the Biuret method. The homogenate was centrifuged at 800 × *g *for 10 min. The resulting supernatant was centrifuged at 8,500 × *g *for 10 min. The supernatant was discarded, and the pellet was diluted in cold isolation buffer and centrifuged at 8,500 × *g *for 10 min three times. The final mitochondria pellet was resuspended in a minimal volume, and aliquots were stored at -80°C until use in enzyme assays. All procedures were conducted at 4°C.

Unless otherwise indicated, the standard incubation medium had the following composition: 100 mM NaCl, 5 mM sodium-potassium phosphate buffer (pH 7.4), 10 mM Tris-HCl buffer (pH 7.4), and 10 mM MgCl_2_.

The respiratory substrates used were 5 mM potassium glutamate plus 2.5 mM potassium malate and 5.0 mM potassium succinate plus 4 μM rotenone.

### Oxygen consumption

Oxygen consumption was measured using a Clark-type electrode (Hansatech Instruments Ltd., using software OXIGRAPH version 1.10; Norfolk, UK) in a 2 mL glass chamber equipped with magnetic stirring.

The reaction was started by the addition of 6 mg mitochondrial protein to 2 mL standard medium containing rotenone 5 μM, oligomycin 1.3 mM, nigericin 100 pmol/mg protein, succinate 10 mM, ADP 200 μM, and glutamate/malate 5/2.5 mM. Finally, it was stabilized for 1 min at 30°C. Respiration rates are given in nAtom-gram oxygen/mg·min. Phosphorylating respiration (state 3) was initiated by addition of 200 nmol ADP/mg protein. Phosphorylation efficiency (ADP/O ratio) was calculated from the added amount of ADP and total amount of oxygen consumed during state 3. The state 4 is obtained with all substrates but ADP.

The ratio between state 3 rate and state 4 rate is called the respiratory control ratio (RCR), and indicates the tightness of the coupling between respiration and phosphorylation. With isolated mitochondria the coupling is not perfect, probably as a result of mechanical damage during the isolation procedure. Typical RCR values range from 3 to 10, varying with the substrate and the quality of the preparation. Coupling is thought to be better *in vivo *but may still not achieve 100%.

### Flow cytometry analysis

Gated mitochondrial population was chosen by flow cytometry, based on forward scatter and side scatter within mitochondria samples, after obtaining one clear mitochondria population.

#### Mitochondrial transmembrane electrical potential (MMP)

MMP (ΔΨ) was measured by the lipophilic cationic fluorescent probe Rh-123 (Molecular Probes Inc., Eugene, OR), a fluorescent derivative of uncharged dihydroRh-123, according to previous studies [4,5,12]. A mitochondrial suspension (50 μg/mL) was incubated with the same respiratory substrates used in oxygen uptake for 1 min at room temperature, and after adding Rh-123 (260 nM) and incubating it for another minute. After incubation, suspensions were immediately analyzed by flow cytometry. The values of the fluorescence (FL) substrates were normalized to the value obtained with the uncoupler carbonylcyamide-m-chlorophenylhydrazone.

#### Rate of intramitochondrial reactive oxygen species (ROS) generation

The rate of ROS generation from mitochondria was measured after the formation of Rh-123 using the cytometry method performed by O'Connor [12] with a small modification. A mitochondrial suspension (100 μg/mL) was incubated with 0.82 nM dihydro Rh-123 and 7 U/mL horseradish peroxidase for 5 min at room temperature. The values of the FL substrates were normalized to the value obtained without peroxidase and adding the uncoupler CCCP. H_2_O_2 _(1 mM) was used with the positive control.

After incubation, the suspensions were immediately analyzed. Gated mitochondrial population was chosen by flow cytometry, based on forward scatter and side scatter within mitochondria samples.

Cytofluorometric analysis was performed using a flow cytometer EPICS XL (Beckman Coulter, Inc., Fullerton, CA) equipped with a single 488 nm argon laser (15 mW). Green FL was detected with a wide-band filter for Rh-123 centred in 525 ± 20 nm (FL1). A standard cytogram based on the measurement of right angle scatter *vs*. forward angle scatter was defined to eliminate cellular debris and aggregates. A minimum of 10,000 mitochondria per sample was acquired in list mode and analyzed with System II version 3.0 Software (Beckman Coulter).

### Activities of mitochondrial complexes

Mitochondrial suspensions were thawed and diluted with potassium phosphate. Activities of the respiratory chain enzymes were measured at 37°C in Cobas Mira (ABXMicro, Mannheim, Germany).

#### Measurements of cytochrome oxidase activity

Cytochrome oxidase activity was measured according to the method described by Cortese *et al*. [16]. Mitochondria were resuspended in the medium containing (in mM) 220 mannitol, 70 sucrose, 2.5 K_2_HPO_4_, 2.5 MgCl_2_, and 0.5 EDTA. Antimycin A was then added to block mitochondrial respiration through complex III. Reaction was started by adding ascorbate/*N, N, N*',*N*'-tetramethyl-*p*-phenylenediamine as an electron donor.

#### Complex V, ATPase (EC 3.6.1.34.)

The activity was assayed by coupling the reaction to the pyruvate kinase and lactate dehydrogenase systems, and measuring reduced nicotinamide adenine dinucleotide (NADH) oxidation at 340 nm. The assay system contained Tris-HCl buffer 65 mM (pH 7.5), sucrose 300 mM, MgCl2 4.75 mM, ATP 4 mM, NADH 0.4 mM, phosphoenolpyruvate 0.6 mM, potassium cyanide 5 mM, pyruvate kinase 700 U/mL, and lactate dehydrogenase 1000 U/mL.

### Assessment of "proton leak": the relationship between respiration rate and MMP (ΔΨ)

Mitochondrial proton leak was calculated from respiration rates and MMP expressing the ratio of protons for each oxygen atom consumed [13-15]. The rate of proton leak across the inner mitochondrial membrane is a function of the driving force (membrane potential) and increases disproportionately with membrane potential.

Titration of membrane potential and state 4 oxygen consumption by respiratory inhibitors were performed simultaneously in separate vessels at 30°C. Nigericin was added to collapse the pH difference across the mitochondrial inner membrane and, thus, ΔΨ had the value of the proton motive force (Δp). Reactions were started by the addition of 3 mg mitochondrial protein/mL standard medium containing also 3 mM rotenone, 1.3 mM oligomycin, nigericin (100 pmol/mg protein), and 5 mM succinate. The addition of inhibitors was begun when the maximum value of the potential became stable (after ~2-3 min). When succinate was used as the substrate, the titration was performed with malonate (K/salt) from 0-13 mM; at the end of each membrane potential trace, the zero point was determined by addition of CCCP 1 mM. Rates of respiration during the titration with inhibitors were measured with a Clark-type oxygen electrode, and membrane potential with a flow cytometer simultaneously with the measurements of membrane potential.

### Inhibition of Adenine Nucleotide Translocase (ANT) by Atractyloside (Atr)

To establish the optimal concentration of Atr (Calbiochem-Novabiochem, San Diego, CA) needed for ANT inhibition, the efficiency of Atr was first examined in its classical role, *i.e*. for its ability to inhibit oxidative phosphorylation. For analysis, increasing Atr concentrations (50-200 pmol/mg mitochondrial protein) were used until complete inhibition of oxygen consumption was obtained [9,18,19].

### Oxidative damage and total antioxidant status (TAS) in isolated mitochondria

Lipid hydroperoxides (LOOHs) were assessed in isolated mitochondria as previously described by Arab and Steghens [20], and adapted for Cobas Mira (600 nm wavelength) and mitochondria suspensions. Briefly, orange xylenol (180 μL-167 μM) was added to 25 μL sample. The first optic reading was obtained before the addition of iron gluconate (45 μL-833 μM). LOOH was calculated using a standard curve of tert-butyl hydroperoxide, and LOOH levels were expressed as nmol/mg mitochondrial protein. Intraassay and interassay coefficients were 3 and 8%, respectively.

TAS, as total enzymatic and nonenzymatic antioxidant capability, was evaluated in isolated mitochondria by a colorimetric assay (Randox Laboratories Ltd., Ardmore, Crumlin, UK) using the following principle: 2,2'-azino-di-('3-ethylbenzthiazoline sulfonate) was incubated with a peroxidase (metmyoglobin) and H_2_O_2 _to produce the radical cation 2,2'-azino-di-('3-ethylbenzthiazoline sulfonate)^·+^. This has a relatively stable blue-green colour, which is measured at 600 nm. Antioxidants in the added sample cause suppression of this colour production to a degree that is proportional to their concentration [21,22].

### Assay for Caspase-3 and 9-Associated Activity

The cytoplasm and nuclear fractions were obtained by cell fractionation. Briefly, tissue was disrupted and treated with lysis buffer (800 μL) containing 10 mM HEPES, pH 7.9, 10 mM KCl, 0.1 mM ethylenediaminetetraacetic acid (EDTA), 0.1 mM EGTA, 5 μg/mL aprotinin, 10 μg/mL leupeptin, 0.5 mM phenylmethylsulfonyl fluoride, 1 mM ditiothreitol (DTT), and 0.6% Nonidet NP-40 for 10 min on ice. Afterward, samples were homogenized and centrifuged at 15,000 g for 3 min at 4 °C. Aliquots of the supernatant (cytoplasm) were stored at -80 °C until use for the measurement caspase-3 activation. The pellet (nuclear fraction) was discarded. The caspase-3-associated activity in the sample (25 μg) was measured using N-acetyl-Asp-Glu-Val-Asp-7-amino-4-trifluoromethy coumarin (Ac-DEVD-AFC, Bachem AG, Bubendorf, Switzerland) (100 μM) in caspase-incubating buffer (50 mM HEPES pH 7.5, 100 mM NaCl, 10% sucrose, 0.1% CHAPS, 1 mM EDTA, and 5 mM DTT) up to 100 μL of total volume. The fluorescence of the sample (Ex = 400, and Em = 505) was recorded using a GENios Microplate Reader (TECAN, Salzburg, Austria).

### Statistical analysis

Data are expressed as means ± sem. Statistical significance was estimated with the paired or unpaired *t *test as appropriate. A *P *value lower than 0.05 was considered significant (*p < 0.05 vs yCO and ^&^p < 0.05 vs O). All analyses were performed using the SPSS version 15.0 (SPSS, Inc., Chicago, IL) statistical package.

## Results

The mitochondrial dysfunction in aging rats was previously characterized [5] in isolated hepatic mitochondria.

### Effect of low doses of IGF-II on Mitochondrial Membrane Potential (MMP)

The MMP, which is considered a good marker of mitochondrial function, was monitored by FL quenching of Rh-123 in mitochondria from the livers of rats under different conditions: the resting state 4 (with all substrates but ADP); the active state 3 (with ADP); and with oligomycin, which deactivates ATPase showing the conditions of maximum intramitochondrial negativity.

Table 1 summarizes the MMP values, expressed as arbitrary units (AU), in the three experimental groups, when succinate was used as substrate. According to previous data [5], a reduction of MMP was observed in untreated aging rats compared with young controls, which IGF-II therapy was able to restore to similar values to those found in young controls, as IGF-I replacement therapy had reached [4,5]. No changes were observed using glutamate/malate as substrates (see Additional File 1, Table S1).

**Table 1:** Mitochondrial Membrane Potential (expressed as arbitrary units of fluorescence, AUF) in isolated liver mitochondria from the three experimental groups, using succinate as substrate.

	Young controls (yCO) (n = 6)	Untreated old rats (O) (n = 6)	Old rats treated with IGF-II O+IGF-II (n = 6)
Succinate (State 4)	191.90 ± 18.05	160.55 ± 16.15^a^	188.60 ± 19.00^b^
+ ADP (State 3)	133.95 ± 13.30	125.40 ± 17.10	131.40 ± 11.40
+ Oligomycin	217.55 ± 17.10	158.65 ± 17.10^a^	223.85 ± 14.25^b^

Values are mean ± SEM. *P*, ns yCO group *vs*. O+IGF-II group in all conditions.

^a ^*P *< 0.05 vs yCO

^b ^*P *< 0.05 vs O

#### Mitochondrial oxygen consumption

Table 2 shows oxygen consumption under different conditions and Respiratory Control Ratios (RCRs) in mitochondria from the three experimental groups, when succinate was used as substrate. Untreated old rats (O group) showed higher values of oxygen consumption compared with young controls, but no significant differences were found between yCO and O+IGF-II groups. Interestingly, mitochondria from old rats treated with IGF-II expended significantly lower amounts of oxygen compared with untreated old animals (*P *< 0.05) with a significantly better efficiency because MMP returned to values similar to those found in young controls, whereas O group showed a depletion of MMP as is described in Table 1 and in a preliminary study [6]. However, when glutamate/malate were used as substrates, no significant changes were found (Suppl. Table 1).

**Table 2:** Oxygen consumption by mitochondria from the three experimental groups, using succinate as substrate.

	Young controls (yCO) (n = 6)	Untreated old rats (O) (n = 6)	Old rats treated with IGF-II O+IGF-II (n = 6)
State 4 (nAgO·mg^-1^·min^-1^)	27.55 ± 2.85	19.95 ± 1.90	23.00 ± 2.00
State 3 (nAgO·mg^-1^·min^-1^)	88.35 ± 6.65	62.70 ± 5.70^a^	74.00 ± 15.00
RCR	3.30 ± 0.80	3.10 ± 0.50	3.20 ± 0.90
ADP/Oxygen	1.71 ± 0.19	1.05 ± 0.28^a^	2.01 ± 0.41^b^

Values are mean ± SEM. ADP/Oxygen expresses oxidative phosphorylation: ATP produced by oxygen molecule consumed. RCR = Respiratory Control Ratio (ratio State 3/State 4).

^a ^*P <*0.05 *vs*. yCO group

^b ^*P *< 0.05 *vs*. O group

In addition, the ratio ADP/Oxygen (ADP/O) expressing oxidative phosphorylation as ATP produced by oxygen molecule consumed, was significantly reduced in mitochondria from O group (*P <*0.05 *vs*. yCO and O+IGF-II groups), when either succinate (Table 2) or glutamate/malate (Suppl. Table 1) were used as substrates, whereas old rats treated with low doses of IGF-II showed similar values to those found in young controls.

No significant differences were found among the three experimental groups in RCRs (state 3 to state 4).

#### Proton leak rates

The rate of proton leak across the inner mitochondrial membrane is a function of the driving force (membrane potential) and increases disproportionately with membrane potential [13,14]. Proton leak rates express "proton escape" into mitochondrial matrix contributing to dissipation of the MMP in pathological conditions.

**Figure 1:**
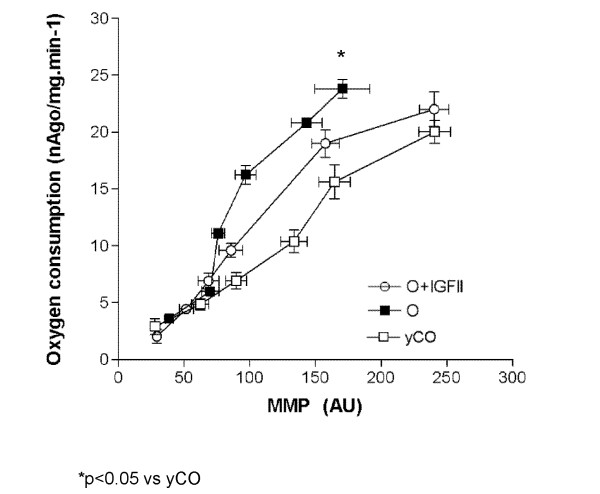
**Proton leak curves expressed by oxygen consumption (nAgO·mg^-1^·min^-1^) and Mitochondrial Membrane Potential (MMP, in arbitrary units) in state 4 (without ADP)**. Proton leak rates express proton "escape" into mitochondrial matrix contributing to the dissipation of the MMP under pathological conditions. Mitochondria from untreated aging rats needed to consume more oxygen to reach the same value of MMP compared with young controls and old rats treated with IGF-II.

Figure 1 shows the proton leak curves in the three experimental groups, expressed by oxygen consumption (nAgO·mg^-1^·min^-1^) at a given MMP in state 4 (without ADP). Mitochondria from untreated old rats needed to consume more oxygen to reach the same MMP values as compared to young controls. As Figure 1 express in mitochondria from old animals treated with IGF-II the "proton escape" was reduced suggesting a more effective utilization of the oxygen leading to a suitable proton gradient.

#### Intramitochondrial Reactive Oxygen Species (ROS) production

**Figure 2:**
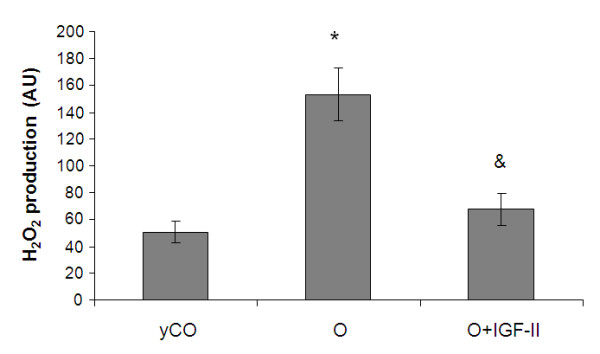
**Intramitochondrial H_2_O_2 _production in isolated mitochondria from the three experimental groups**.

Figure 2 shows the intramitochondrial ROS production in isolated mitochondria from the three experimental groups. Mitochondria from untreated aging rats showed a significant increase of ROS generation compared with mitochondria from young controls and O+IGF-II.

All these data prove an incorrect use of oxygen by mitochondria from untreated old rats, which do not achieve the suitable proton gradient. Consequently the MMP results insufficient for the activity of the ATP synthase.

#### Activities of cytochrome oxidase and ATP synthase complexes

Cytochrome oxidase activity (nAgO·mg^-1^·min^-1^) expressed as oxygen consumption in this complex was significantly reduced in O group compared with young controls (yCO: 65.45 ± 3.90 *vs*. O: 46.10 ± 5.75; *P <*0.05). However, no differences were found between yCO group and old rats treated with IGF-II (O+IGF-II: 61.48 ± 7.10, *P = *not significant *vs*. yCO and *P <*0.05 *vs*. O group).

ATPase activity (expressed as μmol ATP produced per molecule of Oxygen consumed) was significantly reduced in untreated aging rats (O: 0.13 ± 0.01 vs yCO: 0.18 ± 0.01, *P *< 0.05). However, there were no significant differences between yCO and O+IGF-II groups in complex V activity (O+IGF-II: 0.20 ± 0.02, *P *< 0.05 vs O group), according to preliminary data [6].

### Estimation of the vulnerability to pore opening by Atractyloside (Atr)

#### Blockage of oxygen consumption by Atr

**Figure 3:**
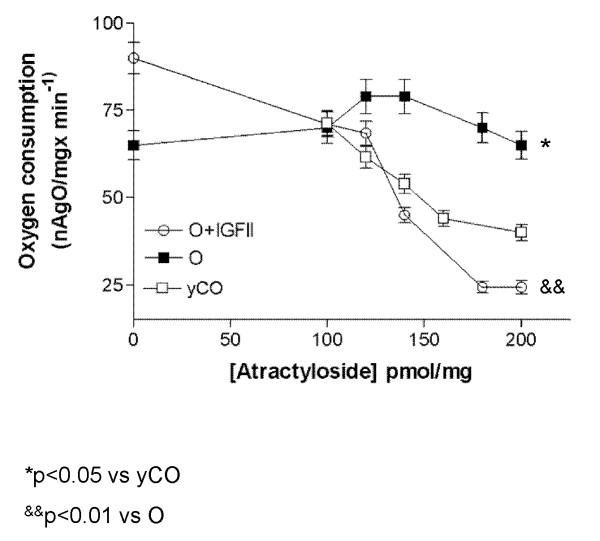
**Blockage of oxygen consumption by Atractyloside (Atr)**. In normal conditions, Atr competes with ADP in ANT blocking oxygen consumption. In this case, Atr was not able to block oxygen consumption in mitochondria from untreated aging rats, suggesting an uncoupling of the ANT probably caused by oxidation of ANT-thiol groups [18,19]. In this condition of mitochondrial oxidative damage, respiration is Atr insensitive.

In physiological conditions Atr competes with ADP in ANT blocking mitochondrial respiration. Full inhibition of the oxygen consumption induced by addition of ADP was obtained in liver mitochondria from young controls at a concentration of 150 pmol/mg Atr: see Figure 3.

In this environment of increased intracellular ROS in old rats, ANT can be oxidized [18,19], leading to an eventually uncoupling of this transporter. In this condition, mitochondrial respiration becomes Atr insensitive. Consistently with this hypothesis, our study show that Atr did not inhibit respiration in mitochondria from untreated aging rats (O group) (Figure 3). Interestingly, Atr inhibition was close to young controls in mitochondria from O+IGF-II showing a normal ANT coupling. In this group (O+IGF-II) as shown in Figure 3, full inhibition of the oxygen consumption was obtained at a concentration of 200 pmol/mg Atr.

### Mitochondrial oxidative damage and Total Antioxidant Status (TAS) in isolated liver mitochondria

**Figure 4:**
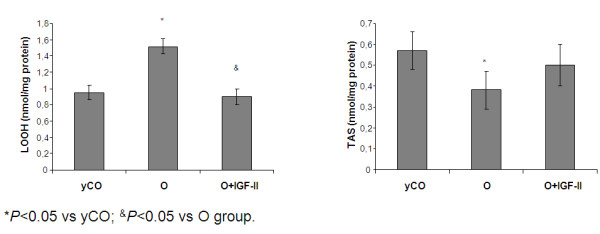
**Lipid oxidative damage and Total Antioxidant Status (TAS) in isolated mitochondria from the three experimental groups**.

Figure 4 shows intramitochondrial oxidative damage, using lipid hydroperoxides (LOOHs) as markers [20-22], and total antioxidant capability of isolated mitochondria [21,22]. Mitochondria from untreated old rats showed an increase in oxidative damage and a reduction in TAS compared with young controls. IGF-II therapy was able to improve both parameters.

### Effect of IGF-II on caspase 3 and caspase 9 activity

**Figure 5:**
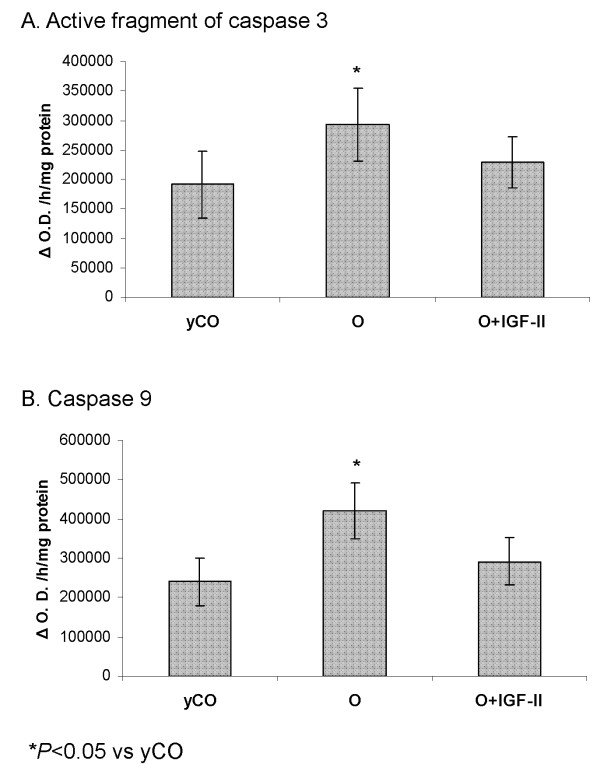
**Assays for caspases 3 and 9 associated activity**.

Assays for caspase 3 associated activity showed a significant increase of caspase 3 activation in untreated aging rats compared with young controls (Figure 5). However, a reduction in the expression of the active fragment of caspase 3 was observed in old animals treated with IGF-II.

Caspase 9 showed the same pattern with an increased activity in untreated aging rats compared with young controls (Figure 5; *P <*0.05). Again, IGF-II treatment induced a significant reduction of the activity of caspase 9 proving a diminution of this apoptotic pathway.

## Discussion

The impact of the reduced activity of GH/IGFs axis in age-related changes is not fully understood. It has been demonstrated that IGF-I replacement therapy reduces the impact of age in rats [4,5], improving glucose and lipid metabolisms, increasing testosterone levels and serum total antioxidant capability and reducing oxidative damage in brain and liver associated with a normalization of antioxidant enzyme activities and mitochondrial function.

These beneficial effects of IGF-I may have been due to suppressing endogenous GH release. Current studies in our laboratory are designed to prove this mechanism.

Understanding that aging is mainly a condition of IGFs deficiency, more than GH, we have recently reported that the exogenous administration of IGF-II induces similar effects of IGF-I in aging rats, without increasing testosterone levels [6]. Therefore, these data allow us to confirm that the neuroprotective and hepatoprotective actions are owed to specific properties of IGFs.

In this context, since mitochondria are one of the most important cellular targets of IGF-I, the present study analyzed the effects of IGF-II therapy on hepatic mitochondria from old animals.

Results in this paper showed that mitochondrial dysfunction leading to apoptosis (by caspase activation) was normalized by IGF-II therapy, at low doses. In fact, IGF-II treatment during 30 days recovered mitochondrial oxidative damage, mitochondrial proton gradient (resulting in an increase of MMP) and ATP synthesis and reduced free radical generation by mitochondria, proton leak rate and the vulnerability for pore opening in ANT which was associated to a reduction of caspase activation compared with untreated old rats.

We had previously characterized mitochondrial dysfunction in aging rats [5]. The observed reduction of MMP with an increased generation of H_2_O_2 _suggests that oxygen is wasted by damaged mitochondria producing ROS instead of a normal proton gradient that is the driving force of ATP synthase [14]. IGF-II therapy was able to reduce mitochondrial membrane damage and restore all parameters of mitochondrial function as IGF-I replacement therapy.

Together, these data suggest an extramitochondrial protection of mitochondria by IGFs, which is not fully established. Previously, we reported that low doses of IGF-I restored the expression of the serine protease inhibitor 2 (α1- antichymiotripsinogen) in cirrhotic rats [23], which could contribute to the outlined mitochondrial protection. In agreement with the results in this paper, it has been reported that IGF-I and II have antiapoptotic properties [24-27].

The main finding in this work was that IGF-II at low doses acts as an analogous of IGF-I inducing cell resistance to apoptosis by oxidative stress through mitochondrial protection leaded to ATP production. IGF-II therapy resulted -as IGF-I replacement therapy- in an increment of ATP synthesis. Interestingly, several beneficial effects of IGF-II in aging [6] could be related to an increased ATP availability similar to those described for IGF-I therapy [4,5], in accordance with Sonntag WE *et al *[28]. This mechanism could explain, at least in part, a significant amount of evidence that have been accumulated during the last years indicating that IGF-I and IGF-II are potent neuronal mitogens and neurotrophic factors [29-35], and more recently the reported action of IGF-II administration in the enhancing memory retention and preventing forgetting [36], suggesting this hormone as a possible novel target for cognitive enhancement therapies.

Another point that deserves particular mention is that IGF-II treatment improved significantly lipid metabolism, diminishing cholesterol and triglycerides and increasing free fatty acids circulating levels [6]. Since fatty acids are synthesized at mitochondria, this result is consistent with both the mentioned normalization of mitochondrial function, and previously reported findings by Liang G and col. [32].

## Conclusion

In conclusion, results in this paper reinforce seriously this concept: aging is mainly a condition of IGFs deficiency, in which mitochondrial dysfunction is one of the most relevant endpoint as an intracellular source of free radicals perpetuating oxidative cellular damage and causing ATP depletion. This work provides new evidence regarding the impact of IGFs declination in aging, clearly suggesting a strong clinical relevance since IGF-II therapy reduces age-related side effects in rats without increasing testosterone levels, potentetially worsening diseases such as prostate hypertrophy or neoplasia.

## List of Abbreviations

ANT: adenine nucleotide translocase; AUF: arbitrary units of fluorescence; bw: body weight; EC: Enzyme Commission of the International Union of Biochemistry; IGF: Insulin-Like Growth Factor; MDA: malondialdehyde; MMP: mitochondrial membrane potential; ns: not significant; O: untreated old rats; O+IGF-II: aging rats treated with IGF-II; PCC: protein carbonyl content; RH123: rhodamine 123 dye; ROS: reactive oxygen species; S: sensitivity; TAS: total antioxidant status; yCO: young controls.

## Competing interests

The authors declare that they have no competing interests. These results have been registered as P200601523.

## Authors' contributions

MG-F, JEP and IS performed the research; LG analyzed the data; and IC-C designed the research, carried out the in vivo protocol and wrote the paper.

All authors have read and approved the final manuscript.

## Supplementary Material

Additional file 1 **Mitochondrial Membrane Potential (expressed as arbitrary units of fluorescence, AUF) and oxygen consumption in isolated liver mitochondria from the three experimental groups, using Glutamate/Malate as substrates**. Click here for file
