# Supplementary material for: Liver mitochondrial dysfunction is reverted by insulin-like growth factor II (IGF-II) in aging rats
Source: J Transl Med. 2011 Jul 28;9:123. doi: 10.1186/1479-5876-9-123 (PMC3162510; doi:10.1186/1479-5876-9-123)
Supplement: Additional file 1 — Mitochondrial Membrane Potential (expressed as arbitrary units of fluorescence, AUF) and oxygen consumption in isolated liver mitochondria from the three experimental groups, using Glutamate/Malate as substrates. [file 1479-5876-9-123-S1.DOC]

**Supplemental Table 1.** Mitochondrial Membrane Potential (expressed as arbitrary units of fluorescence, AUF) and oxygen consumption in isolated liver mitochondria from the three experimental groups, using Glutamate/Malate as substrates.

|  | | **Young controls (yCO)** (n= 6) | | | **Untreated old rats (O)**  (n= 6) | | **Old rats treated with IGF-II O+IGF-II** (n= 6) | | |  |
| --- | --- | --- | --- | --- | --- | --- | --- | --- | --- | --- |
| MMP  State 4 | | 166.25±16.20 | | | 170.30±11.00 | | 180.10±12.55 | | |  |
| + ADP (State 3) | | 131.10±15.75 | | | 133.70±10.85 | | 148.00±18.65 | | |  |
| Oxygen consumption |  | |  | | |  | | |  | |
| State 4  (nAgO·mg^-1^·min^-1^) | 11.45±1.05 | | | 13.35±0.95 | | | | 13.05±1.75 | | |
| State 3  (nAgO·mg^-1^·min^-1^) | 40.85±10.70 | | | 48.45±11.45 | | | | 42.35±9.00 | | |
| RCR | 3.50±0.50 | | | 3.70±0.50 | | | | 3.10±1.00 | | |
| ADP/Oxygen | 2.32±0.16 | | | 1.49±0.35 ^a^ | | | | 2.46±0.61 ^b^ | | |
| Values are mean±SEM. ADP/Oxygen expresses oxidative phosphorylation: ATP produced by oxygen molecule consumed. RCR = Respiratory Control Ratio (ratio State 3 / State 4).  ^a^ *P<*0.05 *vs*. yCO group.  ^b^ *P*<0.05 *vs*. O group | | | | | | | | | |  |
